# Past, Present, and Future of Japanese Encephalitis

**DOI:** 10.3201/eid1508.090149

**Published:** 2009-08

**Authors:** Susan L. Hills, Deborah C. Phillips

**Affiliations:** PATH, Seattle, WA, USA

**Keywords:** Encephalitis, JE, SA 14-14-2, Japan, vaccine, vaccination, viruses, response, letter

**To the Editor:** We are writing in response to the perspective on Japanese encephalitis (JE) by Erlanger et al. (*1*). Growing awareness is encouraging, yet because JE is a largely neglected disease, information is often contradictory or not readily available. We would like to supplement the authors’ review with clarification on available vaccines and actions countries are taking to evaluate and control JE.

There is room for improvement or expansion on collecting and reporting JE surveillance data. However, as vaccine availability increases, many countries are eager to determine the impact of JE and to make informed decisions on immunization programs. For example, surveillance in Indonesia from 2005 through 2006 confirmed human cases throughout the country (*2*). In Cambodia, JE surveillance commenced in 2006, and an immunization program is being planned (*2*). Regional JE laboratory networks established by the World Health Organization are also helping countries gather this information by strengthening diagnostic capacity.

Cambodia plans to introduce the live, attenuated SA 14-14-2 vaccine from China’s Chengdu Institute of Biological Products. This vaccine has recently become internationally available and is increasingly replacing the inactivated, mouse brain–derived vaccine in Asia. A single dose of the SA 14-14-2 vaccine demonstrated 96% efficacy after 5 years (*3*), and the Institute’s commitment to an affordable price for developing countries has broadened accessibility (*4*). The government of India introduced the SA 14-14-2 vaccine in 2006, and nearly 50 million children 1–15 years of age have been reached through vaccination campaigns and routine immunization. The vaccine also is available through public programs or private markets in China, Nepal, South Korea, Sri Lanka, and Thailand.

JE vaccine candidates in late-stage development for children include a live, attenuated chimeric virus vaccine and an inactivated, Vero cell–derived vaccine, each based on the SA 14-14-2 virus strain. Additionally, 2 inactivated, Vero–cell derived vaccines based on the Beijing-1 strain are being developed in Japan (*5*).

New vaccine development, along with progress in surveillance and immunization, offers promise for sustainable control of clinical JE. To achieve this, global partners are working together to develop a strategic plan for JE control by 2015 (*6*).

## References

[1] Erlanger TE, Weiss S, Keiser J, Utzinger J, Wiedenmayer K. Past, present, and future of Japanese encephalitis. Emerg Infect Dis. 2009;15:1–7. 10.3201/eid1501.080311 19116041 PMC2660690

[2] World Health Organization. Third Biregional Meeting on Control of Japanese Encephalitis; 2007 Apr 26–27; Ho Chi Minh City, Vietnam. Manila: World Health Organization Regional Office for the Western Pacific; 2007 [cited 2009 Jan 15]. Available from http://www.wpro.who.int/NR/rdonlyres/50129D1D-E9B3-4707-A62E-0A541DBC3032/0/MTGRPT_JEBireg3.pdf

[3] Tandan JB, Ohrr H, Sohn YM, Yoksan S, Ji M, Nam CM, Single dose of SA 14-14-2 vaccine provides long-term protection against Japanese encephalitis: a case–control study in Nepalese children 5 years after immunization. Vaccine. 2007;25:5041–5. 10.1016/j.vaccine.2007.04.052 17521781

[4] World Health Organization. Sixteenth Meeting of the Technical Advisory Group on Immunization and Vaccine Preventable Diseases in the Western Pacific Region; 2006 Jun 20–22; Manila, Philippines. Manila: World Health Organization Regional Office for the Western Pacific; 2006 [cited 2009 Jan 15]. Available from http://www.wpro.who.int/NR/rdonlyres/1B75A8B9-2F00-4559-9A1F-C55C24A69304/0/MTGRPT_TAG16.pdf

[5] Beasley DWC, Lewthwaite P, Solomon T. Current use and development of vaccines for Japanese encephalitis. Expert Opin Biol Ther. 2008;8:95–106. 10.1517/14712598.8.1.95 18081539

[6] Elias C, Okwo-Bele J, Fischer M. A strategic plan for Japanese encephalitis control by 2015. Lancet Infect Dis. 2009;9:7. 10.1016/S1473-3099(08)70290-1 19095192

